# In search of quality indicators for Down syndrome healthcare: a scoping review

**DOI:** 10.1186/s12913-017-2228-x

**Published:** 2017-04-18

**Authors:** Francine A. van den Driessen Mareeuw, Mirjam I. Hollegien, Antonia M. W. Coppus, Diana M. J. Delnoij, Esther de Vries

**Affiliations:** 1 Tranzo, Scientific Center for Care and Welfare, Faculty of Social and Behavioural Sciences, Tilburg University, PO Box 90153 (T-329), 5000 LE Tilburg, The Netherlands; 2 Department of Paediatrics, Jeroen Bosch Hospital, ´s-Hertogenbosch, The Netherlands; 3 Dichterbij, Center for the Intellectually Disabled, Gennep, The Netherlands; 4 Department for Primary and Community Care, Radboud University Medical Center, Nijmegen, The Netherlands; 5 Institute for Health Care Quality, National Health Care Institute, Diemen, The Netherlands; 6 Severinus, Centre for the Intellectually Disabled, Veldhoven, The Netherlands

**Keywords:** Down syndrome, Intellectual disability, Quality of health care, Quality indicators, Quality measures, Integrated delivery of health care

## Abstract

**Background:**

The medical care chain around Down syndrome (DS) is complex, with many multidisciplinary challenges. The current quality of care is unknown. Outcome-oriented quality indicators have the potential to improve medical practice and evaluate whether innovations are successful. This is particularly interesting for the evolving care for people with DS and intellectual disabilities (ID). The aim of this study was to identify existing indicators for medical DS care, by reviewing the literature.

**Methods:**

We systematically searched six databases (PubMed, EMBASE, Web of Science, CINAHL, PsycINFO, Google Scholar) for studies concerning the development and implementation of quality indicators for DS and/or ID care, published until February 1^st^ 2015. The scoping review method was used, including systematic data extraction and stakeholder consultation.

**Results:**

We identified 13 studies concerning quality indicators for ID care that obtained data originating from questionnaires (patient/family/staff), medical files and/or national databases. We did not find any indicator sets specifically for DS care. Consulted stakeholders did not come up with additional indicator sets. Existing indicators for ID care predominantly focus on support services. Indicators in care for people with ID targeting medical care are scarce. Of the 70 indicators within the 13 indicator sets, 10% are structure indicators, 34% process, 32% outcome and 24% mixed. Ten of the 13 sets include indicators on the WHO quality dimensions ‘patient-centeredness’, ‘effectiveness’ and ‘efficiency’ of care. ‘Accessibility’ is covered by nine sets, ‘equitability’ by six, and ‘safety’ by four. Most studies developed indicators in a multidisciplinary manner in a joint effort with all relevant stakeholders; some used focus groups to include people with ID.

**Conclusion:**

To our knowledge, this is the first review that searched for studies on quality indicators in DS care. Hence, the study contributes to existing knowledge on DS care as well as on measuring quality of care. Future research should address the development of a compact set of quality indicators for the DS care chain as a whole. Indicators should preferably be patient-centred and outcome-oriented, including user perspectives, while developed in a multidisciplinary way to achieve successful implementation.

**Electronic supplementary material:**

The online version of this article (doi:10.1186/s12913-017-2228-x) contains supplementary material, which is available to authorized users.

## Background

Down syndrome (DS), or (partial) trisomy 21, is the most prevalent chromosomal anomaly among new-borns with intellectual disabilities. The overall prevalence throughout the world is about 10 per 10000 new-borns [1–3]. DS is associated with a broad variety of age-related medical problems, ranging from congenital heart disease to dementia to recurrent respiratory infections [1–3]. The care chain around a person with DS is challenging and complex, involving numerous professionals [3–5]. This requires coordination of care and adequate age- and service-related transitions [4, 5].

Initiatives arise to improve the DS care. Skotko *et al.* (2013) describe how a DS specialty clinic can identify and address many healthcare needs of children and adolescents with DS beyond the provision of primary care [6]. In the Netherlands, numerous paediatric outpatient clinics now organise such multidisciplinary team appointments, including a visit to the paediatrician, physiotherapist, ENT (earn-nose-throat)-specialist and others, all on the same day. For adults with DS in the Netherlands, healthcare is less organised, although some 18+ teams are being set up [7]. Internationally, difficulties are identified in care transition (from paediatric to adult care) and in persistent use of paediatric care by DS adults [8]. An achievement towards higher quality care for DS has been the development of guidelines [9, 10]. In general, health checks are increasingly developed in the care for people with intellectual disabilities (ID) [11, 12]. However, the quality of existing initiatives and the extent to which healthcare professionals adhere to existing guidelines is unclear [13, 14]. More insight is needed into the care that is delivered to people with DS, in terms of types of care, its quality and its effect on clinical outcomes [14]. Quality indicators (also known as quality measures [15, 16]) can provide this insight. They have the potential to structure the development of multidisciplinary teams, improve clinical decisions and guide organisational reform [17]. This study aimed to review existing data on quality indicators for DS care, including both clinical and organisational aspects, and to identify existing indicator sets.

Evaluating quality of healthcare (by using indicators) starts with defining ‘quality of healthcare’. About half a century ago (1966) Donabedian formulated the frequently used framework that distinguishes three healthcare components: *structure, process and outcome* [17]. Accordingly, the quality of each of these ‘care components’ can be measured by structure, process or outcome *indicators*. Structure indicators assess the availability of the right facilities, such as staff, supplies, policies and protocols, but also the financial basis, e.g. insurance [18]. Process indicators assess whether “good” medical care, according to current evidence/knowledge, has been applied [17]. Care processes are actions that take place between a patient and care provider, i.e. technical interventions (e.g. measuring blood pressure) or interpersonal interactions (e.g. doctor-patient communication) [19]. In practice, process indicators are often operationalized as adherence to guidelines, but they could also include general assumptions like access to and timeliness of services, and coordination and continuation of care. Outcomes are the consequences of delivered care and the actual results of healthcare interventions, also expressed as the five Ds: death, disease, discomfort, disability and dissatisfaction [20]. Contributions of healthcare to the patient’s quality and length of life may also be qualified as outcomes of healthcare [21, 22]. Outcome indicators have the potential to evaluate care cycles as a whole instead of single processes by itself [23]. Traditionally, measurement instruments (such as indicator sets) for quality of healthcare contain all three types of indicators [24].

Next to these three types of healthcare components, several *quality dimensions* of healthcare are defined. The World Health Organisation (2006) defines six dimensions of quality of care, i.e. care being effective, efficient, accessible, patient-centred, equitable and safe [25]. When it comes to *integrated care*, other quality dimensions should be considered as well, such as continuity and adequate transitions between care organisations [26].

Additionally, quality of care can be assessed at different levels, e.g. at the level of single providers, departments, hospitals or at the level of care chains as a whole: the combined efforts of all care providers together [27]. In the end, it is this care chain that delivers the total package of care to the patient, resulting in the final outcome [23]. Addressing the care chain as a whole in quality evaluation is quite challenging, because so many organisations and people are involved [23].

In order to contribute to quality improvement, indicators measuring quality of healthcare should themselves be of good quality, e.g. evidence based, and they should measure what they are designed to measure. An instrument that can be used as a manual to develop indicators is the AIRE instrument (Appraisal of Indicators through Research and Evaluation) [27]. In addition, AIRE can be used as a checklist to appraise the quality of indicators [28].

This study aims to review existing quality indicators for the DS care chain (for both children and adults with DS). We focus on the following research question:

*Which indicators are available to assess the clinical and organisational quality of medical DS healthcare?*

More specifically:

1. *Which indicator sets are available and which indicators do they contain?*
  - *Which components and levels of care are covered by these indicators?*
  - *Of which type (structure, process or outcome) are these indicators?*
2. *What is the quality of these indicator sets?*
  - *Which dimensions of quality are covered by the sets?*
  - *How have the sets been developed and implemented?*
  - *What can be said about other quality aspects of the sets?*

## Methods

A scoping study was carried out to map available indicator sets of healthcare for people with DS. A scoping study (or scoping review) is a specific type of literature review that may be used to examine research activity in a certain field of study, assess the usefulness of conducting a full systematic review, summarise research findings, or identify gaps in literature [29, 30]. Scoping studies are often conducted when little research has been done on the topic studied and a specific research question cannot be formulated [30, 31]. In an attempt to ascertain rigorousness and transparency, Arksey and O’Mally (2005) constructed a framework for conducting scoping studies [29]. The framework consists of five stages: 1) identifying the research question; 2) identifying relevant studies (search strategy); 3) selecting the studies; 4) charting the data (data extraction); 5) collating, summarising and reporting the results; and 6) (optional) consultation of stakeholders, resulting in suggestions for additional references and views [29, 30]. We followed these stages.

### Search strategy

The databases of PubMed, EMBASE, Web of Science, CINAHL, PsycINFO and Google Scholar were systematically searched for articles published until February 1, 2015 (no starting date). These six databases were selected together with a librarian to cover a wide range of biomedical and psychological literature from the perspective of different healthcare professionals (physicians, psychologists and nurses). The first group of search terms consisted of synonyms for people with DS. The second group of search terms comprised outcomes to target quality indicators, including quality management, quality improvement and benchmarking. Since results for only DS(−synonyms) were very scarce, the first group of search terms was broadened by adding search terms for (synonyms for) people with intellectual disabilities (ID) (Table 1). Search strategies were similar for each database, except for Google Scholar, which required a more narrowly defined search, since the entry fields did not accept as many search terms as the entry fields of the other databases.

**Table 1:** Search strategy

Population:	Outcomes:
1 Intellectual Disability 2 Mentally Disabled Persons 3 Developmental Disabilities 4 Down Syndrome 5 Developmental disorder* 6 Mental deficien* 7 Mental retard* *8 Down’s syndrome* *9 Trisomy 21* 10 (1 OR 2 OR 3 OR 4 OR 5 OR 6 OR 7 OR 8 OR 9) (Google Scholar: 1 OR 2 OR 3 OR 4 OR 5 OR 6 OR 7) 19 (NOT) Pregnancy	11 Quality Indicators, Health Care 12 Quality Improvement 13 Total Quality Management 14 Benchmarking 15 Clinical indicator* 16 Quality measure* 17 Quality assessment* 18 (11 OR 12 OR 13 OR 14 OR 15 OR 16 OR 17) (Google Scholar: 11 OR 16)

Combining search term groups: 10 AND 18 NOT 19

This strategy is related to the PubMed search. Very similar versions were used to search EMBASE, Web of Science, CINAHL, PsycINFO and Google Scholar, but adapted for the specific search terms used in these databases, if available. The search terms printed in italics are not MeSH-terms. All MeSH Terms were also searched as free text in all databases as title/abstract

### Study selection

**Fig. 1:**
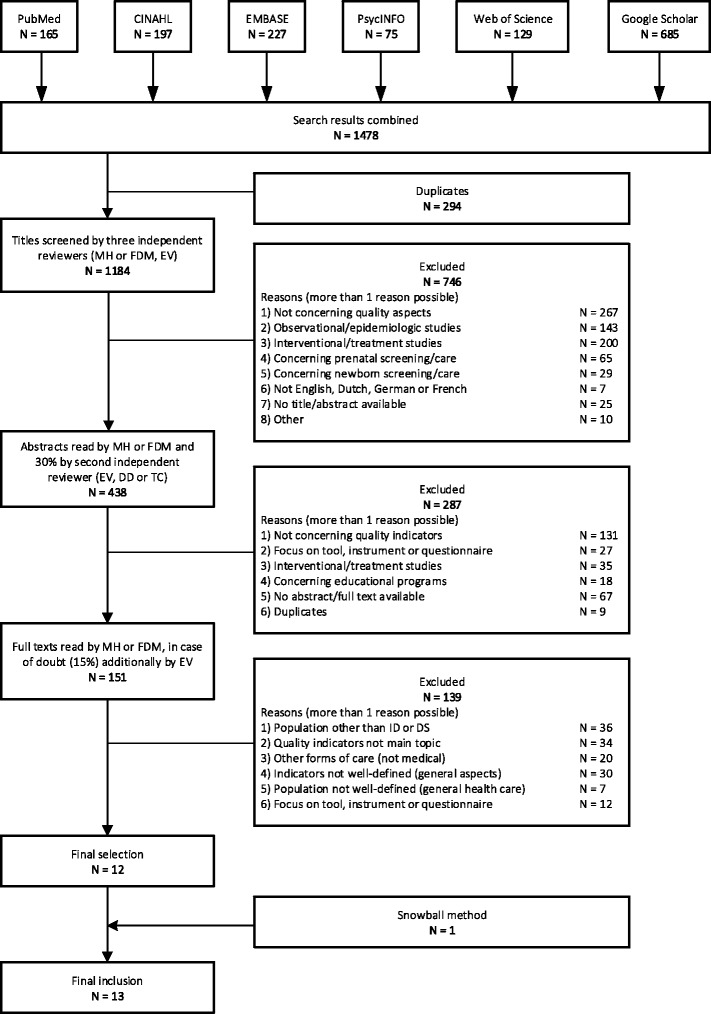
Flow chart of selection process. Number of studies found per database, title selection, abstract selection, full text selection, and snowball method resulting in final inclusion of 13 studies

Figure 1 shows the selection process in a flowchart. Specific inclusion and exclusion criteria are listed in Table 2. In the first selection phase, duplicates were removed, and two independent reviewers (MH or FDM, and EV) screened all titles. Titles were included in the next selection phase when they concerned quality aspects of healthcare for chronic conditions (comparable to DS care). This review focuses on the care chain for individuals with DS (or ID) from birth to end-of-life. Therefore, we excluded articles concerning prenatal screening. In the next selection phase, abstracts were screened based on more narrow criteria: focus on the development, implementation, application or evaluation of indicators for measuring quality of healthcare. MH and FDM selected all abstracts (partly by MH, partly by FDM) and a random selection of 30% of all abstracts was screened by a second reviewer (EV, DD, AC, each 10%), which resulted in 26% differences in interpretation. For instance, one abstract mentioned ‘Quality deficiencies’; FDM concluded from this that the study was not about indicators, whereas DD thought quality deficiencies could be another word for quality indicators: the study was selected. Another study was not selected, because AC doubted about inclusion and FDM interpreted that the study was not about indicators for healthcare. Discussion between the reviewers resolved all differences, which resulted in 100% agreement about in- or exclusion. MH and FDM reviewed full texts (partly by MH, partly by FDM). In case of any doubt, EV also reviewed the articles and a third and fourth reviewer (DD and AC) was consulted in case of disagreement. In this final phase, quality indicators had to be the main topic, well defined (as well as the population they applied to) and more specifically concerning medical healthcare, as opposed to e.g. residential care. A snowball method was applied in order to find additional studies: Reference lists of the selected studies were screened for additional relevant studies. If titles mentioned in the reference lists suggested relevant information (on development, implementation or evaluation of indicators), these studies were retrieved and, based on full texts, FDM assessed whether the studies provided additional information. If the studies provided information about additional indicator sets and matched inclusion criteria, these studies were included. If snowball-studies in turn mentioned additional indicator sets in the text, corresponding references were searched too and included if relevant (this happened once).

**Table 2:** Inclusion and exclusion criteria

Inclusion criteria: • Studies concerning the development, implementation, application or evaluation of (structure, process or outcome) indicators for measuring quality of (chronic) medical healthcare for people with Down syndrome or intellectual disabilities as the main topic • Studies where specific quality indicators are well-defined including the population they apply to • All kinds of scientific publications: journal articles, theses, books, etc.
Exclusion criteria: • Studies where quality indicators itself are not the main topic • Studies not concerning medical care, but other forms of care (e.g. residential care) • Studies concerning general aspects of quality indicators (specific indicators are not well-defined) • Studies concerning quality indicators of general healthcare (specific population is not described) • Studies primarily focusing on the development of a tool, instrument or questionnaire without the purpose of being an indicator for measuring quality of healthcare • Studies concerning prenatal or new-born screening/care • No abstract/full text available • Written in a language that no one in the research team masters (i.e. not English, Dutch, French, German)

### Data extraction

As the included studies did not always provide enough information to be able to answer our research questions, additional information about the indicator sets was sought. This was done by looking on websites of the organisations who developed the indicator sets and by entering the name of the indicator set in Google and Google scholar.

We extracted data concerning general information about the indicator sets (name of indicator set, author, year, country, target population and organisational context) and about quality domains covered. With the additional information, we were able to assess the indicators in the sets in terms of type (structure, process, or outcome) and quality using the AIRE instrument (mentioned previously). Two researchers (FDM plus EV, DD or AC) appraised each indicator set. The AIRE instrument results in a score for each of its four categories: 1) Aim, relevance and organisational context; 2) Involvement of stakeholders; 3) Scientific evidence; and 4) Further underpinning, formulation and use. For each category, the reviewers need to score several items on a 4-point Likert-scale: 1 meaning not at all agree and 4 meaning very much agree. If no information was available about an item, this was scored as 1. Table 3 provides an overview of the four categories of the instrument and of the items per category.

**Table 3:** AIRE instrument categories and items per category [27]

Categories	Items
1) Aim, relevance and organisational context	- Aim is clearly defined, - Topic relevance is specified, - Organisational configuration (level) is specified, - Quality domain is specified, - Type and size of care process the indicator set applies to is defined.
2) Involvement of stakeholders	- Relevant healthcare professionals are involved in developing the set, - Relevant other are involved, - The indicator set is formally established (or owned), e.g. by a patient or professional association.
3) Scientific evidence	- Underpinning evidence for the set is systematically searched, - The set is based on a guideline, - The Used evidence is qualitatively good.
4) Further underpinning, formulation and use	- Denominator and numerator are clearly described, - Target population is specifically and clearly defined, - A risk adjustment strategy (for different patient groups) is present, - Validity of the set is proven or argued, - Reliability of the set is proven or argued, - Power of the set is proven or argued, - The set is tested in practice, - The effort needed for data collection is taken into account, - The set includes an instruction for interpretation of the results.

One researcher (FDM) assessed the type of the indicators, as the definition of the types was clear and all indicators could be easily attributed to one of the three types. Some indicators were very broadly defined and were therefore classified as ‘mixed’, covering information about two or more of the types. For each set, the percentages of the indicator types were calculated, after which the percentages per type were added up in order to provide an idea of relative distribution of indicator types for all the indicators in the sets.

### Consultation exercise

Twenty representatives from the healthcare perspective (professionals providing different sorts of healthcare to people with DS in the Netherlands) and three from the healthcare receivers (board members of a leading Down syndrome association in the Netherlands) were asked (by e-mail) to review the list of selected studies and check whether they missed studies or indicator sets. We also asked them about their opinions concerning indicator sets for DS care in general. Four representatives (from the professionals group) did not review the identified studies and indicator sets because of time constraints and/or lack of interest in the topic.

## Results

The literature search yielded 1184 studies (see Fig. 1). No studies specific for DS care were found. Thirteen studies were selected for final inclusion: they contained quality indicators for medical healthcare in people with ID (see Table 5, second column). Consultation of stakeholders did not result in additional studies or indicator sets. All stakeholders agreed that developing indicators for medical care for people with DS would be worthwhile for improving quality or transparency (see Table 4).

**Table 4:** Answers of stakeholders

	Number of times mentioned by stakeholders (*N* = 19)
Why are indicators for DS relevant?	
*To define care*	8
*For coordination*	7
*For quality improvement*	8
*For comparability of care providers*	14
*To check availability*	3
Additional studies?	
*No*	11
*Yes but not about indicators*	8

### Research question 1: Which indicator sets are available and which indicators do they contain?

Thirteen different indicator sets were identified (Table 5), five of which originate from the UK, four from the USA, one from Canada, one from Ireland, one from Sweden, and one as a result of a partnership between 13 European countries.

**Table 5:** Overview of identified indicator sets described by selected studies and general information about the sets

Indicator set	Described by selected study	Country of origin/development	Target population	Number of indicators (sub-indicators) and Topics covered by indicators in set	Organisational level	WHO quality domains
1 Ambulatory Care Sensitive Conditions (ACSC) [39, 40]	Glover & Evison, 2013 [41]	Canada	Persons with an intellectual disability	15: “conditions which, given ‘effective management’ at the primary care level, should not normally result in an admission to hospital”	Primary care	Effective, efficient, accessible
2 Hospital Admissions for Ambulatory Care Sensitive Conditions (ACSC) [41]	Glover & Evison, 2013 [41]	UK	People with learning disabilities (LD)	3 (22): Acute conditions, Chronic conditions, immunisable conditions.	National health system of England	Effective, efficient, accessible
3 Healthcare Effectiveness Data and Information Set (HEDIS®) [42–44]	Shireman et al., 2010 [42]	USA	Adults with developmental disabilities with Diabetes	5: HbA1c testing, eye examinations, lipid testing, microalbuminaria screening, primary care visits	National/whole care chain	Effective, patient-centered
4 The Health Equalities Framework (HEF) [45, 46]	Thomas, 2014 [45]	UK	People with learning disabilities (LD)	5 (29): Social indicators, Genetic and biological indicators, Communication difficulties and reduced health literacy indicators, Personal behaviour and lifestyle indicators, Deficiencies in service quality and access indicators	Specialist multidisciplinary learning disability services	Efficient, accessible, patient-centered, equitable, safe
5 Measurement of Processes of Care (MPOC-28) [47, 48]	Granat et al., 2002 [47]	Sweden	Families with children with disabilities	4 (28): Enabling and partnership, General & specific information (given by care provider), Co-ordinated and comprehensive care, Respectful and supportive care	Child habilitation services departments	Efficient, accessible, patient-centered
6 National Core Indicators (NCI) [49–51]	Bradley et al., 2007 [49]	USA	Children and adults with developmental disabilities and their families	5 (94): Individual outcomes (satisfaction, choice and decision making, self-determination, community inclusion, work, relationships), Health welfare and rights (safety, health, medication, wellness, restraints, repsect/rigths), System performance (Sevice coordination, Access, staff stability), Family indicators (choice & control, family outcomes, information & planning, satisfaction, family involvement, community connections, access & support delivery).	Public systems for people with intellectual and developmental disabilities	Accessible, patient-centered, equitable, safe
7 Quality Indicators ~ February 2004 Learning Disabilities (NHS-QIS) [52, 53]	Campbell, 2008 [54] NHS QIS, 2004 [52]	UK, Scotland	Children and adults with learning disabilities in Scotland	6 (60): Involvement of Children and Adults with Learning Disabilities and Their Family Carers through Self-Representation and Independent Advocacy, Promoting Inclusion and Wellbeing, Meeting General Healthcare Needs, Meeting Complex Healthcare Needs, In-patient Services - Daily Life, Planning Services and Partnership Working	National Health System of Scotland	Effective, efficient, accessible, patient-centered, equitable, safe
8 Health indicators for people with intellectual disabilities (Pomona-project) [55, 56]	van Schrojenstein L-de Valk et al., 2007 [56] (snowball)	Europe	People with intellectual disabilities in Europe	4 (18): Demographics, Health status, Determinants of health, Health systems.	European/national	Effective, efficient, patient-centered, equitable
9 Quality indicators for preventive care [57–59]	Coker et al., 2012 [57]	USA	Children aged 10 months to 5 years old who are at risk for developmental delay	4 (14): Parents' Evaluation of Developmental Status, Comprehensive and coordinated care, Family-centered and culturally effective care, medical home.	Preventive care	Effective, efficient, accessible, patient-centered
10 Quality care indicators of diabetes for people with ID [60] [61]	Taggart et al., 2013 [60]	UK	People with intellectual disabilities and diabetes	1(6): HbA1c checked, Lipids/cholesterol, Eye exam, Weight change, Physically active, Attended emergency department related to DM	Diabetes care chain	Effective, efficient, patient-centered
11 Six Core Outcomes: Key Measures of Performance [62–66]	Spears, 2010 [62]	USA	Children with special healthcare needs	6: Shared decision making, Coordinated care, Adequate insurance, Screening for special healthcare needs, Community-based services, Services for transitions.	States' and Territories' service systems	Effective, efficient, accessible, patient-centered
12 Quality and Outcomes Framework Indicators for learning disabilities (QOF) [67–73]	Ashworth, 2012 [67]	UK	People with learning disabilities in the UK	1(2): Learning Disability register, % Patients in register with Down's Syndrome aged 18 and over who have a record of blood TSH in the previous 15 months.	Primary care	Effective, efficient, equitable
13 Quality indicators measuring the quality of the medication use process for people with intellectual disabilities [37, 74]	Flood & Henman, 2014 [37]	Ireland	People ageing with intellectual disabilities	5 (37): Patient experience, access to care, continuity of care, equity, patient safety, effectiveness, appropriateness, assessment.	Medication use process care chain	Effective, accessible, patient-centered, equitable, safe

Out of the 13 identified indicator sets, three have not been specifically developed for people with ID. The three studies describing these sets only evaluated existing indicators in people with ID, by comparison with the general population (no. 9, Quality indicators for preventive care; no. 3, Healthcare Effectiveness Data and Information Set; no. 10, Quality care indicators of diabetes for people with ID). Others adjusted existing sets of indicators to apply them in care for people with ID (no. 1, Ambulatory Care Sensitive Conditions; no. 2, Hospital Admissions for Ambulatory Care Sensitive Conditions; no.5, Measurement of Processes of Care; no. 11, Six Core Outcomes). Three indicator sets have been developed or used for children with, or at risk for, ID, i.e. no. 5 (MPOC-28), no. 9 (Quality indicators for preventive care), and no. 11 (Six core outcomes). An overview of the indicators per set, including their content, can be found as Additional file 1 to this article.

### Research question 1a: Which components and levels of care are covered by the indicators?

The indicator sets cover a large variety of healthcare levels (settings) and topics. The sets predominantly evaluate the presence of facilities/services or the effectuation of care delivery at communicational and organisational levels. Most of the sets include indicators on collaboration, multidisciplinary cooperation, transition and coordination. Five of the identified sets focus on quality of supportive care and services, containing only a subcategory of indicators being applicable to medical care: no. 3 (The Health Equalities Framework, HEF), no. 6 (National Core Indicators, NCI), no. 7 (the NHS quality indicators for Learning Disabilities, NHS-QIS), no. 9 (the Quality indicators for preventive care), and no. 11 (the Six Core Outcomes). Medical care is approached in a general way and specific diseases and/or treatment courses are barely addressed. Indicators on medical topics primarily focus on screening and preventive care. Two sets consider hospitalisation rates as indicators for conditions which, given effective primary care, should not normally result in hospital admission. Their indicators aim to measure access to, and quality of, primary care: no. 1 (Ambulatory Care Sensitive Conditions) and no. 2 (Hospital Admissions for Ambulatory Care Sensitive Conditions). One set, no. 12 (Quality Outcomes Framework, QOF) contains - among others - an indicator named ‘Learning disabilities’, which comprises a measure for a register of patients with learning disabilities and a measure for thyroid disease among people with DS. This is the only set explicitly addressing DS. The QOF indicators have been designed to measure the quality of primary care in Great Britain. Two indicator sets include measures for diabetes care for people with intellectual disabilities (no. 3, Healthcare Effectiveness Data and Information Set; no. 10, Quality care indicators of diabetes for people with ID). Lastly, two sets focus on processes of care: i.e. no. 5 (MPOC-28) concerning processes in child rehabilitation and no. 13 (Quality indicators for medication use process) including indicators for medication use in people with ID.

### Research question 1b: Of which type (structure, process and outcome) are the indicators?

The number of indicators per set varies widely. The thirteen sets together comprise 70 separate indicators, ranging from 2 to 6 indicators per set. Most indicators in turn consist of a number of sub-indicators ranging from 14 to 94. Altogether (regardless of sub-indicators) we identified 6 structure, 21 process, 26 outcome indicators, and 12 indicators measuring a mix of structure-, process-, or outcome-measures. When calculating the percentages of types of indicators per sets, and then adding up the percentages per type, it appeared that 10% of the 70 indicators are structure indicators, 34% process, 32% outcome and 24% mixed. Table 6 presents the distribution of the types of indicators per set.

**Table 6:** Relative and absolute proportion of types of indicators in identified indicator sets

Type of indicator→	Structure	Process	Outcome	Mix
Indicator sets ↓				
1 ACSC CAN	0	0	100% (15)	0
2 ACSC UK	0	0	100% (3)	0
3 HEDIS DM	0	100% (5)	0	0
4 HEF	0	40% (2)	20% (1)	40% (2)^a^
5 MPOC-28	0	100% (4)	0	0
6 NCI	20% (1)	20% (1)	20% (1)	40% (2)^b^
7 NHS-QIS	33% (2)	17% (1)	0	50% (3)^c^
8 POMONA	0	0	75% (3)	25% (1)^d^
9 Preventive care	0	75% (3)	25% (1)	0
10 Diabetes UK	0	0	0	100% (1)^e^
11 Six core outcomes	33% (2)	67% (4)	0	0
12 QOF	50% (1)	0	50% (1)	0
13 Medication use process	0	20% (1)	20% (1)	60% (3)^f^
Total	86 (6)	439 (21)	420 (26)	315 (12)

^a^Mixed indicators consist of a mix of 1) structure & outcome sub-indicators and 2) structure & process sub-indicators

^b^Mixed indicators consist of a mix of 1) structure & process & outcome sub-indicators and 2) structure & process sub-indicators

^c^Mixed indicator consist of a mix of structure & process sub-indicators

^d^Mixed indicator consist of a mix of structure & process sub-indicators

^e^Mixed indicator consist of a mix of process & outcome sub-indicators

^f^Mixed indicators consist of a mix of 1) process & outcome sub-indicators (2x) and 2) process & outcome & structure sub-indicators

### Research question 2: What is the quality of the indicator sets?

**Fig. 2:**
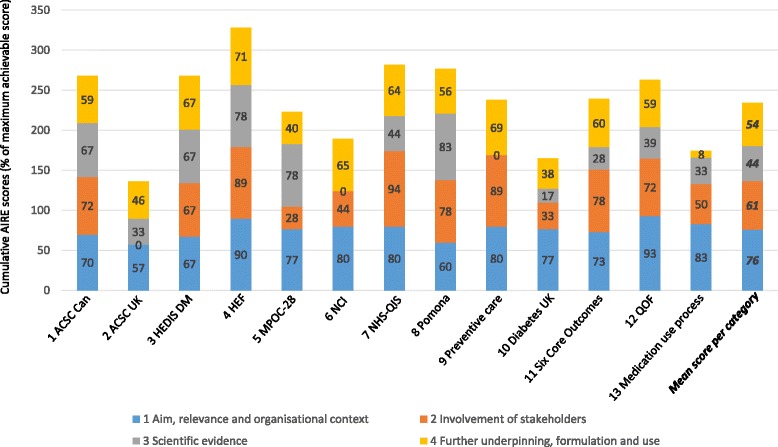
AIRE-scores per set. Scores are calculated as percentage of maximal achievable score. Each colour in a bar reflects the score for an AIRE-score category

The quality of the indicator sets was assessed using the AIRE instrument. The AIRE-scores are presented in Fig. 2.

Although category 1 did not get the highest score in all sets (sets 1, 7, 8, 9, and 11 got a higher score on category 2 and set 5 on category 3), category 1 is the best scoring category on average. All sets have clearly defined the aim and relevance and specify the organisational configuration, type of care, quality dimension on which the indicators apply, and indicate the relevance of the topic. All WHO quality dimensions (effective, efficient, accessible, patient-centred, equitable and safe) are covered (Table 7), although some dimensions are only covered by a small number of sets (e.g. only four indicator sets cover ‘safety’). The domains ‘effective’, ‘efficient’, and ‘patient-centred’ are covered by ten of the sets. This implies that a large part of the indicator sets aim to measure (and improve) these dimensions of care. ‘Accessibility’ is covered by nine sets, ‘equitability’ by six, and ‘safety’ by four.

**Table 7:** Quality dimensions covered by indicator sets, per dimension

Quality dimension →	Effective	Efficient	Accessible	Patient-centered	Equitable	Safe
Indicator sets ↓						
1 ACSC CAN	√	√	√			
2 ACSC UK	√	√	√			
3 HEDIS DM	√			√		
4 HEF		√	√	√	√	√
5 MPOC-28		√	√	√		
6 NCI			√	√	√	√
7 NHS-QIS	√	√	√	√	√	√
8 POMONA	√	√		√	√	
9 Preventive care	√	√	√	√		
10 Diabetes UK	√	√		√		
11 Six core outcomes	√	√	√	√		
12 QOF	√	√			√	
13 Medication use process	√		√	√	√	√
Number of sets covering dimension	10	10	9	10	6	4

In general, there are differences in whether relevant stakeholders have been involved in developing the sets (AIRE-category 2). In most studies, indicators have been developed in a multidisciplinary manner with involvement of the relevant stakeholders. These stakeholders involve general practitioners, paediatricians, psychologists, social workers, direct care staff, researchers, policy makers, managers and/or family members. In most cases, the actual content of the multidisciplinary team is not clearly described. Two studies have been using focus groups to include people with ID in the development process (Atkinson et al. 2013, van Schrojenstein Lantman-de Valk et al. 2007). Other ways of obtaining data for the development of indicators include Delphi studies, web-based applications, on-site observations, staff questionnaires, medical file recordings, financial registrations, content of protocols and/or national databases.

The evidence base of the sets, category 3, provided the lowest scores, though some sets score quite high (no. 1, 3, 4, 5 and 8).

Finally, category 4 (Further underpinning, formulation and use) covers a large variety of indicator characteristics (see Table 3) and the score for this category differs between the sets. Some of the sets do not contain indicators with a numerator and denominator, e.g. the two sets on diabetes care contain the indicator ‘patient’s HbA1c is checked’. Furthermore, some sets clearly report how validity and reliability have been assured, while others do not contain any information on that. The same is true for the power of the sets (the extent to which an indicator is sensible to measure changes). Almost all sets have to some extent been implemented and tested in practice. However, some sets have only been implemented and tested once, while others have been in use for many years. Data collection of the indicator sets also varied. For three of the sets, data collection methods are not (yet) specified (sets 1, 4 and 13). Six of the sets (sets 5, 6, 8, 9, 10, and 11) collect data through telephone surveys, postal questionnaires or face-to-face interviews with people with ID or their representatives. Three sets use existing registrations for obtaining data (2, 3, and 7). For one set (12), general practices have to score points on several topics, it is unclear whether this is done through a questionnaire or existing registrations.

## Discussion

### Summary of results

We reviewed the literature to identify indicators that assess the clinical and organisational quality of medical care for people with DS. Only one of the found studies described an indicator set containing one single indicator on thyroid disease among people with DS; the other studies were not about DS care. Therefore, we have chosen to search for quality indicators in care for people with ID that could be applicable in DS care. We have found that quality indicators in care for people with ID targeting medical care, instead of supportive care and services, were scarce. We reviewed to what extent these indicators cover the structure, process and outcome of care. The majority of indicators concern processes of care for performance measurement. Many sets include indicators on coordination, multidisciplinary working and cooperation. The six WHO quality dimensions are well covered by the sets, although ‘safety’ is the least addressed. We also aimed to evaluate the development and implementation of the indicators. Most quality indicators have been developed in a multidisciplinary manner with relevant stakeholders, some using focus groups to include people with ID. Almost all sets have to some extent been implemented and tested in practice. Data collection for the indicators is achieved in multiple ways, such as consumer/family surveys, medical file recordings, and/or national databases. The sets differ in quality aspects, e.g. some authors describe thoroughly how validity and reliability was assured, how sensible the indicators are and what the evidence base is, while others barely address these issues.

### Quality indicators in medical care for people with ID and DS

The most striking finding of the current study is that quality indicators specific for DS care have not been published to date (except for the single set containing one indicator on thyroid disease among people with DS). Moreover, the indicators found for the care for people with ID barely address medical aspects. Generally, people with DS and people with ID have similar health needs [4], which may imply that the identified quality indicators would be applicable in DS care as well. However, people with DS usually have more and many specific comorbidities compared to the general population of people with ID [4]. This urges the need for both medical care that is specifically tailored to the healthcare needs of people with DS and DS specific indicators, which can contribute to the quality of life of people with DS [6]. Indicators for care for people with ID would not be specific enough. DS specific indicators can reveal bottlenecks in the care chain and can lead to the identification of successful interventions and contributors to a specific outcome [23].

The high prevalence of comorbidities among people with DS also requires multidisciplinary collaboration and coordination. Many of the indicator sets found in this study contain indicators for these requirements. They are general concepts that are applicable to different healthcare sectors, regardless of the patient group. Thus, regarding multidisciplinary collaboration and coordination, the identified indicators could be used in a set for healthcare for people with DS.

The six WHO quality dimensions could also be used to define potential indicators [25]. In this study we found that the dimensions ‘effective’, ‘efficient’, and ‘patient-centred’ are predominantly covered (ten out of thirteen), while improvement of care – addressing total care chains – should always be done by paying attention to all the six dimensions [25]. Nonetheless, we believe that ‘equitability’ and ‘patient-centeredness’ should receive special attention in DS. People with DS experience inequality in received healthcare [32]. The comorbidities, communication difficulties caused by intellectual disability, and unusual presentation of common diseases of people with DS require more effort from healthcare professionals to deliver good care [6].

### Structure, process or outcome of care

Of the indicator sets we found in this study, many consist of a large number of process indicators. Outcome indicators also comprise a significant part (although less than process) of the indicators in the sets. The number of structure indicators is the lowest.

Many organisations focus on the assessment of structural aspects and service delivery for performance measurement. They seem to assess results that are easy to reach and easy to measure, with data readily collectable [19, 23, 33, 34]. Structural aspects of care are essential, as they are the basis of the healthcare system. Structure indicators are based on the assumption that given the presence of right physical or staff characteristics, good care automatically results [17]. However, focusing merely on the structural context as an end in itself, may result in overshadowing the initial goal of improving health outcomes for patients [33].

Process indicators are based on *how* healthcare is delivered, e.g. coordination, timeliness, interactions, and *what* interventions take place, e.g. screening or diagnostic tests, treatment etc. Measuring processes has several benefits: they can be measured on a short-term (i.e. directly after care has been delivered), data are easily obtained and differences between organisations are relatively easy to interpret. In general, process indicators are largely based on (the adherence to) guidelines, consisting of recommendations based on current evidence, or best knowledge. Measuring the adherence to guidelines results in important information on the feasibility of recommended care and to some extent, information on care quality. However, standards of best clinical practice are not stable and almost never final [17]. When we solely measure processes we might risk anchoring what is currently known as best practice, which might result in ceasing of innovation [23].

Outcome indicators measure the consequences of delivered care and actual results of healthcare interventions. They reflect whether structural context and processes in single organisations, as well as total care chains [20], actually lead to health benefits. This information on desired, as well as detrimental outcomes may stimulate innovation through the identification of its contributing factors [23]. Outcomes can therefore be interpreted as fundamental measures for quality of healthcare.

### Developing an indicator set for DS

According to the above, development of indicators for medical care should focus on developing outcome indicators. There are however some considerations that should be taken into account. Firstly, stakeholders may have different views on which outcomes are desirable. Whereas survival may be the best scenario in the eyes of a physician, a patient may choose functional status above life expectancy. In addition, change in health-status may not always be the primary goal, especially in long-term care [26], support and processes of care may be of greater importance. Indeed, when evaluating user perspectives on this topic, users primarily seem to focus on processes of care or procedural outputs [24, 26]. As patients are the experts when it comes to their outcomes, it is essential to include people with DS and/or their parents in the process to define what is valuable to them [35]. Their views on quality differ from those of professionals and researchers [26]. Physicians and all other professionals, including healthcare managers, should also be involved, since they might appraise the usefulness and quality of indicators in a different manner [36]. By involving all stakeholders in the development process their conflicting interests can be identified and weighed against each other. We also saw this stakeholder involvement in the development of many of the identified indicator sets. Defining potential quality indicators for DS should thus involve all relevant stakeholders [27, 37] (e.g. general practitioners, paediatricians, psychologists, social workers, direct care staff, researchers, policy makers, managers and family members).

Secondly, another consideration when developing outcome indicators is that before outcomes become manifest, long periods of time may elapse and data will not be readily available [17, 19, 23]. Therefore, long-term measures should be accompanied with intermediate, short-term outcomes [20].

Thirdly, as stated before, multidisciplinary working is of vital importance in medical care for people with DS. Moreover, Callaghan (2006) argues that, especially for people with ID, multidisciplinary collaboration leads to better personal outcomes [38]. This would be a reason for including process indicators, since multidisciplinary working is a typical process aspect of care. On the other hand, as multidisciplinary working leads to personal outcomes, outcome indicators may also be suitable to measure quality of care. In any case, multidisciplinary collaboration should be taken into consideration, whether it is measured by process or outcome indicators.

Fourthly, patient characteristics and environmental factors, e.g. intrinsic motivation or socio-economic status, have an important role in influencing health outcomes as well, beyond the control of individual health professionals [19], not to mention comorbidity. Hence, adjusting for this kind of factors outside the healthcare system that may influence health outcome is important when it comes to interpreting outcomes data [20]. It has to be identified what exactly leads to the result that is measured. Clinical expertise is needed for adequate interpretation, though what the expected outcomes are, is not always known [17].

Finally, when developing indicators one should consider that healthcare systems differ per country or state [19]. Indicators should fit in the care system they apply to. In the Netherlands for example, some DS specific initiatives have been developed. However, specialised care for adults with DS is still scarce [7]. Structural indicators may help in the development of this care, by defining what structural components of care are needed.

To conclude, quality indicators for medical DS care should focus on outcomes, with the above considerations advocating the additional use of some process and structure indicators.

### Strengths and limitations

To our knowledge, this is the first review that searched for studies on quality indicators in DS care. With the use of six different databases, we covered a wide range of scientific publications. Moreover, this review discusses strategies for future development of indicators. The study contributes to existing knowledge on DS care as well as on measuring quality of care for other chronic conditions. A strength of the study is the consultation of relevant stakeholders as a last step of the review, which enabled us to check whether we had missed relevant studies or indicator sets. The fact that no additional indicator sets or studies came up in the stakeholder consultation, shows that we did not miss studies and advocates the quality of this review. Additionally, all stakeholders considered development of quality indicators for care for people with DS relevant, which also indicates the relevance of this study.

This study yielded no indicator sets on medical DS healthcare and the found indicator sets for ID healthcare predominantly focus on non-medical care (e.g. supportive care). This may be the result of including (synonyms for) intellectual disabilities as a search term, which may have put an emphasis on cognitive disability, which is not necessarily related to medical care. Using search terms on for example congenital abnormality or genetic defects might have possibly yielded more medical studies. However, these studies might have been too general and less applicable to DS. As ID is one of the outcomes of DS, we chose to search for studies on ID.

A limitation of the study was that the information of the identified indicator sets was somewhat incomplete. We only searched for information through the internet. Due to this incomplete information, not all items of the AIRE instrument, used to assess the quality, could be scored by the reviewers. Therefore, the low AIRE scores, especially regarding the evidence base of the sets, do not necessarily mean that the evidence base of the sets is not good. The low scores may also be a result of little available information on the sets. Consulting organisations that had developed the indicator sets might have yielded more information. However, the number of items with missing information is small and without the AIRE-scores, we are still able to show information on quality (development, implementation, quality domains).

## Conclusions

This review gives an overview of different strategies for quality measurement. Quality indicators specific for DS care have not been published to date and in the found studies about the care for people with ID medical aspects are barely addressed. Quality indicators can play a major role in improving medical practice and evaluating whether innovations are successful. This is particularly interesting for the evolving DS care, as well as care for people with ID. As illustrated in this review, it is very hard to focus on specific care quality aspects, when approaching such a diverse, large group as ‘people with intellectual disabilities’. Therefore, we recommend focussing on well-defined, DS-specific care chains when developing indicators. Further research activities should include the preparation and development of a compact set of indicators to evaluate and monitor the quality of the DS care chain as a whole. Future indicators should preferably be patient-centred and outcome-oriented, including user perspectives. In order to achieve successful implementation, it is crucial that all care providers support the indicator set, and that all care providers, patients (and/or their parents), and healthcare managers are involved in the process of development.

## Additional file

Additional file 1: Overview of indicator sets with indicators. (DOCX 57 kb)

## Abbreviations

ACSC: Ambulatory Care Sensitive Conditions
AIRE: Appraisal of Indicators Through Research and Evaluation
DS: Down syndrome
HEDIS: Healthcare Effectiveness Data and Information Set ®
HEF: Health Equalities Framework
ID: Intellectual disabilities
MPOC: Measurement of Processes Of Care
NCI: National Core Indicators
NHS-QIS: National Health Services – Quality Improvement Scotland
QOF: Quality and Outcomes Framework (Indicators for learning disabilities)
WHO: World Health Organisation
