# Supplementary material for: In search of quality indicators for Down syndrome healthcare: a scoping review
Source: BMC Health Serv Res. 2017 Apr 18;17:284. doi: 10.1186/s12913-017-2228-x (PMC5395825; doi:10.1186/s12913-017-2228-x)
Supplement: Additional file 1: — Overview of indicator sets with indicators. (DOCX 57 kb) [file 12913_2017_2228_MOESM1_ESM.docx]

**Additional file: Separate indicators per set and topics covered by indicators.** [1-37]

Indicator numbers correspond to the indicator set they belong to. For example: 2.1 means: indicator set number 2, first indicator; 1.0 means: indicator set number one, only indicator in set.

| **No.** | **Indicators and sub-indicators** | **type** |
| --- | --- | --- |
| 1.0 | Ambulatory Care Sensitive Conditions:  Asthma; Angina Pectoris; Congestive heart failure; Gastrointestinal ulcer; Immunization preventable infection; Malignant hypertension; Otitis Media; Neurotic depressive disorders; Dental conditions; Diabetes Mellitus; Pelvic inflammatory disease; Constipation; Gastroesophageal reflux; Epilepsy; Schizophrenic disorders. | Outcome |
| 2.1 | Acute conditions:  Cellulitis; Convulsions and epilepsy; Dehydratation and gastroenteritis; Dental conditions; Ear-nose-throat (ENT) infections; Gangrene; Pelvic inflammatory disease; Perforated/bleeding ulcer; Pyelonephritis; Constipation | Outcome |
| 2.2 | Chronic conditions:  Angina; Asthma; Chronic obstructive pulmonary disease; Congestive heart failure; Diabetes complications; Hypertension; Iron-deficiency anaemia; Nutritional deficiencies; Gastro-oesophageal reflux disease (GORD); Osteoporosis. | Outcome |
| 2.3 | Immunisable conditions:  Influenza and pneumonia; Other vaccine preventable conditions | Outcome |
| 3.0 | Comprehensive Diabetes Care:  HbA1c testing; eye examinations; lipid testing; microalbuminaria screening; primary care visits | Process |
| 4.1 | 1 Social indicators:  Accomodation; Employment, meaningful activities and engagement; Financial support; Social contacts; Additional marginalising factors (such as ethnicity, speech differences); Safeguarding | Structure-Outcome |
| 4.2 | 2 Genetic and biological indicators:  Assessment of physical and mental health needs and health checks; Long Term Condition (LTC) pathways and planned reviews of need; Care Planning / health action planning; Crisis / emergency planning and hospital passports; Medication evaluation; Specialist learning disability service provision | Process |
| 4.3 | 3 Communication difficulties and reduced health literacy indicators:  Poor bodily awareness, reduced pain responses and communication support; Communicating health needs to others; Carers' ability to recognise expressions of needs / pain; Carers' ability to recognise and respond to emerging health problems and / or promote health literacy; Understanding Health Information and Making Choices | Structure- Process |
| 4.4 | 4 Personal behaviour and lifestyle indicators:  Diet and hydration; Exercise; Weight; Substance Use; Sexual Health; Risky Behaviour / Routines | Outcome |
| 4.5 | 5 Deficiencies in service quality and access indicators:  Organisational barriers; Consent; Transition between services; Health screening / promotion; Primary / Secondary Care; Non health services. | Process |
| 5.1 | Enabling and partnership:  Healthcare professional informs parent; trust parent as expert of the child; anticipates concerns; answers questions etc. | Process |
| 5.2 | General and specific information:  Healthcare professional gives information about services in community, about child's disability, therapies, etc. | Process |
| 5.3 | Co-ordinated and comprehensive care:  Healthcare professional looks at the needs of the 'whole' child (e.g. at mental, emotional and social needs), plans together with other health professionals; informs you in time about changes in care; communicates with school, ensures that family receives support. | Process |
| 5.4 | Respectful and supportive care:  Healthcare professional helps parent to feel competent, provides enough time, a caring atmosphere, treats parent respectful. | Process |
| 6.1 | Individual Outcomes:  Satisfaction with, and Choice and Decision-Making regarding housing, daily activities and work; Choice and Decision-Making about daily activities, housing etc.; Self-Determination: Needed and received help with daily activities/budget; Community inclusion; Work; Relationships. | Outcome |
| 6.2 | Health, Welfare, and Rights:  Safety (incidence of serious injuries, mortality, support, feeling safe, victim of crime); Health (health status, received tests and screenings, health status, presence of primary care doctor); Medication; Wellness (healthy habits); Restraints; Respect/Rights (rights are respected; treated with respect by others). | Outcome-process-structure |
| 6.3 | System Performance:  Service Coordination (satisfaction with received help from service coordinators); Access (capable staff; availability of transportation and support/care when needed) | Structure-process |
| 6.4 | Staff Stability:  Continuity of staff presence (vacation rate, trainees, job switches) | Structure |
| 6.5 | Family Indicators:  Choice and Control (Family's control/decision making about budgets; care); Family Outcomes (support for family in caring for their relative); Information and Planning (information about planning care and involvement of family); Satisfaction (of family with care for relative); Community Connections (integration of family in community); Access and Support Delivery (family reported access to and satisfaction with services and support). | Process |
| 7.1 | 1 Involvement of Children and Adults with Learning Disabilities and Their Family Carers through Self-Representation and Independent Advocacy:  Involving people in planning services; in planning care across all services; Policy for access to health records; Complaints procedure; Advocacy (strategy and services are present) | Structure-Process |
| 7.2 | 2 Promoting Inclusion and Wellbeing:  Disability awareness (Disability Discrimination Act; Strategy; Safe Access); Transport; Policy and accessible information on Health promotion and health improvement; Health information and cultural sensitivity; Direct payments to people with ID) | Structure-Process |
| 7.3 | 3 Meeting General Healthcare Needs:  Assessment (of health and capacities); Care plan is present; Primary care and community services (named specialist practitioner, responsive to needs, national screening, monitoring, joint working); specific services for wheelchair and older people are present; General health and hospital services (education for healthcare professionals, advice from specialists; aware of needs; palliative care; specific illnesses) | Structure |
| 7.4 | 4 Meeting Complex Healthcare Needs:  Service integration (specialised & general health services); Transitions (age/service-related); Access to and availability of specialist services (Children/Adults/Complex needs/Challenging or offending behaviours/mental health problems/Autism spectrum dis./Dementia/Profound and multiple impairment/Learning disabilities and epilepsy); | Process |
| 7.5 | 5 In-patient Services - Daily Life:  Environment (plan and accommodation); Privacy and personalisation; Daily life (making own choices) | Process-structure |
| 7.6 | 6 Planning Services and Partnership Working:  Strategic health improvement and needs assessment (strategies); Database developments; Healthcare planning; Hospital closure and service reprovision; Partnership working | Structure |
| 8.1 | Demographics:  Prevalence of ID in population; Living arrangements; Daily occupation; Income/socio-economic status; Life expectancy. | Outcome |
| 8.2 | Health Status:  Epilepsy; Oral Health; Body mass index; Mental Health; Sensory capacities; Mobility. | Outcome |
| 8.3 | Determinants of health:  Physical activity; Challenging behaviour; Psychotropic medication use | Outcome |
| 8.4 | Health Systems:  Hospitalisation and contact with healthcare professionals; Health check; Health promotion; Specific training for physicians | Structure-process |
| 9.1 | Parents' Evaluation of Developmental Status:  Parents have concerns (or not) about their child's learning, development or behavior. | Outcome |
| 9.2 | Comprehensive and coordinated care:  The child had a personal doctor or nurse; usual source of care; parent received needed help with coordination and referrals without problems | Process |
| 9.3 | Medical Home:  a personal doctor or nurse, a usual source of care, family -centered care, care coordination if needed, no problems receiving needed referrals | Process |
| 9.4 | Elicitation of parental developmental concerns and developmental screening:  Healthcare providers asked parents about concerns about child's learning, development or behavior; healthcare provider asks parents to complete an age-appropriate standardised developmental screening tool | Process |
| 10.0 | Quality care indicators of diabetes for people with ID:  HbA1c checked; Lipids/cholesterol; Eye exam; Weight change; Physically active; Attended emergency department related to Diabetes Mellitus | Process-outcome |
| 11.1 | 1 Shared decision making:  Families of CSHCN (children with special healthcare needs) partner in decision-making at all levels and are satisfied with the services they receive | Process |
| 11.2 | 2 Coordinated care:  CSHCN receive coordinated, ongoing, comprehensive care within a medical home (a medical home means a source of ongoing, comprehensive, coordinated, family-centered care in the child's community) | Process |
| 11.3 | 3 Adequate insurance:  Families of CSHCN have adequate private and/or public insurance to pay for the services they need. | Structure |
| 11.4 | 4 Screening for special healthcare needs:  Children are screened early and continuously for special healthcare needs | Process |
| 11.5 | 5 Community-based services:  Community-based services for CSHCN are organised so families can use them easily | Structure |
| 11.6 | 6 Services for transitions:  Youth with special healthcare needs receive the services necessary to make transitions to all aspects of adult life, including adult healthcare, work and independence. | Process |
| 12.1 | Learning disabilities register:  The contractor establishes and maintains a register of patients with learning disabilities. | Structure |
| 12.2 | Thyroid disease among people with DS:  Percentage of patients on the Learning Disability register with Down's Syndrome aged 18 and over who have a record of blood TSH in the previous 15 months (excluding those who are on the thyroid disease register) | Outcome |
| 13.1 | Crucial QIs:  Medication review, General health review, Restrictive practice, Excessive dose, Anti-psychotic medication, Gradual dose reduction, Dementia anti-psychotic medication. | Process |
| 13.2 | Grade 1 QIs:  Multiple medication use/polypharmacy, Anti-cholinergic medication, Anti-depressant medication, Psychotropic medications, Psychotropic/neuroleptic side effects, Dysphagia, Insomnia treatment and sleep behavior, Dementia cholinesterase inhibitors - anticholinergic medication. | Process-Outcome |
| 13.3 | Grade 2 QI:  Geriatric syndromes | Outcome |
| 13.4 | Grade 3 QIs:  Informational transfer, Communication, Medication reconciliation, Residential care, Pharmaceutical care/pharmacist, Non-pharmaceutical care/pharmacist, External environment, Dementia cholinesterase inhibitors, Dental-oral health, Pain, Infections, As requires 'PRN' psychotropic medications, Psychotropic medication physical side effects, Adverse drug reactions. | Process-Outcome-Structure |
| 13.5 | Grade 4 QIs:  Acute behavior, Advocate, Covert administraion of medication, Inter-intra-class psychotropic multiple medication use/polypharmacy, Anti-epileptic medications, Off Label psychotropic medications, Gastro-intestinal disorders, Autism spectrum disorder. | Process-Outcome |

**References**

1. National Core Indicators™. <http://www.nationalcoreindicators.org/> (2015). Accessed 16 Jul 2015.

2. About National Core Indicators. <http://www.nationalcoreindicators.org/about/> (2015). Accessed 16 July 2015.

3. HEDIS® Measure Development Process. <https://www.ncqa.org/Portals/0/HEDISQM/Measure_Development.pdf> (year unknown). Accessed 22 July 2015.

4. HEDIS® Measures. <http://www.ncqa.org/HEDISQualityMeasurement/HEDISMeasures.aspx> (year unknown). Accessed 20 Jul 2015.

5. Ashworth M. The Quality and Outcomes Framework in the United Kingdom: Indicators in Transition. J Ambul Care Manage. 2012; 35(3):200-5.

6. Atkinson D, Boulter P, Hebron C, Moulster G, Giraud-Saunders A, Turner S: The Health Equalities Framework (HEF) An outcomes framework based on the determinants of health inequalities. Bath, United Kingdom: National Develpment Team for Inclusion, Improving Health and Lives: Learning Disabilities Observatory, UK Learning Disability Consultant Nurse Network; 2013.

7. Balogh R, Brownell M, Ouellette-Kuntz H, Colantonio A. Hospitalisation rates for ambulatory care sensitive conditions for persons with and without an intellectual disability--a population perspective. J Intellectual Disabil Res. 2010; 54(9):820-32.

8. Balogh RS, Ouellette-Kuntz H, Brownell M, Colantonio A. Ambulatory Care Sensitive Conditions in Persons with an Intellectual Disability – Development of a Consensus. J Appl Res Intelect Disabil. 2011; 24(2):150-8.

9. Blumberg SJ, Foster EB, Skalland BJ, Chowdhury SR, O'Connor KS: Design and Operation of the National Survey of Children’s Health, 2007. Hyattsville, Maryland, USA: National Center for Health Statistics, DEPARTMENT OF HEALTH AND HUMAN SERVICES, Centers for Disease Control and Prevention 2007.

10. Blumberg SJ, Welch EM, Chowdhury SR, Upchurch HL, Parker EK, Skalland BJ: Design and Operation of the National Survey of Children with Special Health Care Needs, 2005–2006. Washington DC, USA: National Center for Health Statistics. Vital and Health Statistics; 2008.

11. Bradley VJ, Moseley C. National core indicators: Ten years of collaborative performance measurement. Intellect Dev Disabil. 2007; 45(5):354-8.

12. Campbell M. The importance of good quality services for people with complex health needs. Br J Learn Disabil. 2008; 36(1):32-7.

13. Coker TR, Shaikh Y, Chung PJ. Parent-reported quality of preventive care for children at-risk for developmental delay. Acad Pediatr. 2012; 12(5):384-90.

14. Cunningham BJ, Rosenbaum PL. Measure of processes of care: a review of 20 years of research. Dev Med Child Neurol. 2014; 56(5):445-52.

15. NATIONAL INSTITUTE FOR HEALTH AND CLINICAL EXCELLENCE: QUALITY AND OUTCOMES FRAMEWORK (QOF) INDICATOR DEVELOPMENT PROGRAMME Briefing paper. London, United Kingdom: NATIONAL INSTITUTE FOR HEALTH AND CLINICAL EXCELLENCE; 2009.

16. NATIONAL INSTITUTE FOR HEALTH AND CLINICAL EXCELLENCE: Primary Care Quality and Outcomes Framework Indicator Advisory Committee recommendations. London, United Kingdom: NATIONAL INSTITUTE FOR HEALTH AND CLINICAL EXCELLENCE; 2010.

17. NATIONAL INSTITUTE FOR HEALTH AND CLINICAL EXCELLENCE: QUALITY AND OUTCOMES FRAMEWORK (QOF) INDICATOR DEVELOPMENT PROGRAMME Draft QOF indicator guidance. London, United Kingdom: NATIONAL INSTITUTE FOR HEALTH AND CLINICAL EXCELLENCE; 2013.

18. NATIONAL INSTITUTE FOR HEALTH AND CLINICAL EXCELLENCE. Learning Disabilities. <https://www.nice.org.uk/standards-and-indicators/qofindicators?categories=3901&page=1> (2016). Accessed 4 Jan 2016.

19. NATIONAL INSTITUTE FOR HEALTH AND CLINICAL EXCELLENCE. Standards and Indicators. <http://www.nice.org.uk/standards-and-indicators> (2016). Accessed 4 Jan 2016.

20. NATIONAL INSTITUTE FOR HEALTH AND CLINICAL EXCELLENCE. How we develop the NICE Indicator Menu for the QOF. <http://www.nice.org.uk/standards-and-indicators/How-we-develop-QOF> (2016). Accessed 4 Jan 2016.

21. Flood B, Henman M. Building quality indicators for medication use in people aging with intellectual disabilities and behaviour disorders. Int J Dev Disabil. 2014, doi:2047387714Y. 0000000061.

22. Flood B, Henman MC. Case study: hidden complexity of medicines use: information provided by a person with intellectual disability and diabetes to a pharmacist. Br J Learn Disabil. 2015, doi:10.1111/bld.12121.

23. Glover G, Evison F: Hospital Admissions That Should Not Happen. Lancaster, United Kingdom: Improving Health and Lives: Learning Disabilities Observatory; 2013.

24. Granat T, Lagander B, Borjesson MC. Parental participation in the habilitation process - Evaluation from a user perspective. Child Care Health Dev. 2002; 28(6):459-67.

25. Data Resource Center for Child & Adolescent Health. Guide to Topics & Questions Asked. In: National Survey of Children's Health (NSCH)*.* 2007.

26. Data Resource Center for Child & Adolescent Health. Guide to Topics & Questions Asked. In: National Survey of Children with Special Health Care Needs (NS-CSHCN), 2009-2010*.* 2009.

27. POMONA II Research group. POMONA II: Gezondheidswijzer voor mensen met verstandelijke beperkingen. [POMONA II: health guideline for people with intellectual disabilities]. In: Een indicatorset gebruiken [Using an indicator]*.* Edited by Project 2004130 European Commission: Directorate C – Public health and risk assessment: POMONA II; 2006.

28. NHS Quality Improvement Scotland: Quality Indicators Learning Disabilities. Edinburgh, United Kingdom: NHS Quality Improvement Scotland; 2004.

29. NHS Quality Improvement Scotland: National Overview Learning Disability Services. Edinburgh, United Kingdom: NHS Quality Improvement Scotland; 2006.

30. Shireman TI, Reichard A, Nazir N, Backes JM, Greiner KA. Quality of diabetes care for adults with developmental disabilities. Disabil Health J. 2010; 3(3):179-85.

31. Spears AP. The Healthy People 2010 outcomes for the care of children with special health care needs: an effective national policy for meeting mental health care needs? Matern Child Health J. 2010; 14(3):401-11.

32. Strickland BB, van Dyck PC, Kogan MD, Lauver C, Blumberg SJ, Bethell CD, Newacheck PW. Assessing and ensuring a comprehensive system of services for children with special health care needs: a public health approach. Am J Public Health. 2011; 101(2):224-31.

33. Taggart L, Coates V, Truesdale-Kennedy M. Management and quality indicators of diabetes mellitus in people with intellectual disabilities. J Intellectual Disabil Res. 2013; 57(12):1152-63.

34. Thomas B. Improving care for people with learning disabilities. Nursing Standard. 2014; 28(21):31.

35. U.S. Department of Health and Human Services, Health Resources and Services Administration, Maternal and Child Health Bureau: The National Survey of Children with Special Health Care Needs Chartbook 2005–2006. Rockville, Maryland, USA: U.S. Department of Health and Human Services; 2007.

36. Diabetes UK: Diabetes 15 Healthcare Essentials. London, United Kingdom: Diabetes UK; 2014.

37. van Schrojenstein Lantman-de Valk H, Linehan C, Kerr M, Noonan-Walsh P. Developing health indicators for people with intellectual disabilities. The method of the Pomona project. J Intellectual Disabil Res. 2007; 51(6):427-34.
